# Supplementary material for: Identifying multicellular spatiotemporal organization of cells with SpaceFlow
Source: Nat Commun. 2022 Jul 14;13:4076. doi: 10.1038/s41467-022-31739-w (PMC9283532; doi:10.1038/s41467-022-31739-w)
Supplement: Supplementary file 1 — Supplementary Information [file 41467_2022_31739_MOESM1_ESM.pdf]

Supplementary Information

Identifying Multicellular Spatiotemporal Organization of Cells with SpaceFlow

Honglei Ren[1], Benjamin L. Walker[1,2], Zixuan Cang[3], Qing Nie[1,2,4,*]

[1] The NSF-Simons Center for Multiscale Cell Fate Research, University of California Irvine, Irvine, CA, 92627, USA

[2] Department of Mathematics, University of California Irvine, Irvine, CA, 92627, USA

[3] Department of Mathematics, North Carolina State University, Raleigh, NC 27695.

[4] Department of Developmental and Cell Biology, University of California Irvine, Irvine, CA, 92627, USA

[*] Corresponding Author: qnie@uci.edu

This file includes the following subsections:

- Supplementary Figures
- Supplementary Tables

Supplementary Figures

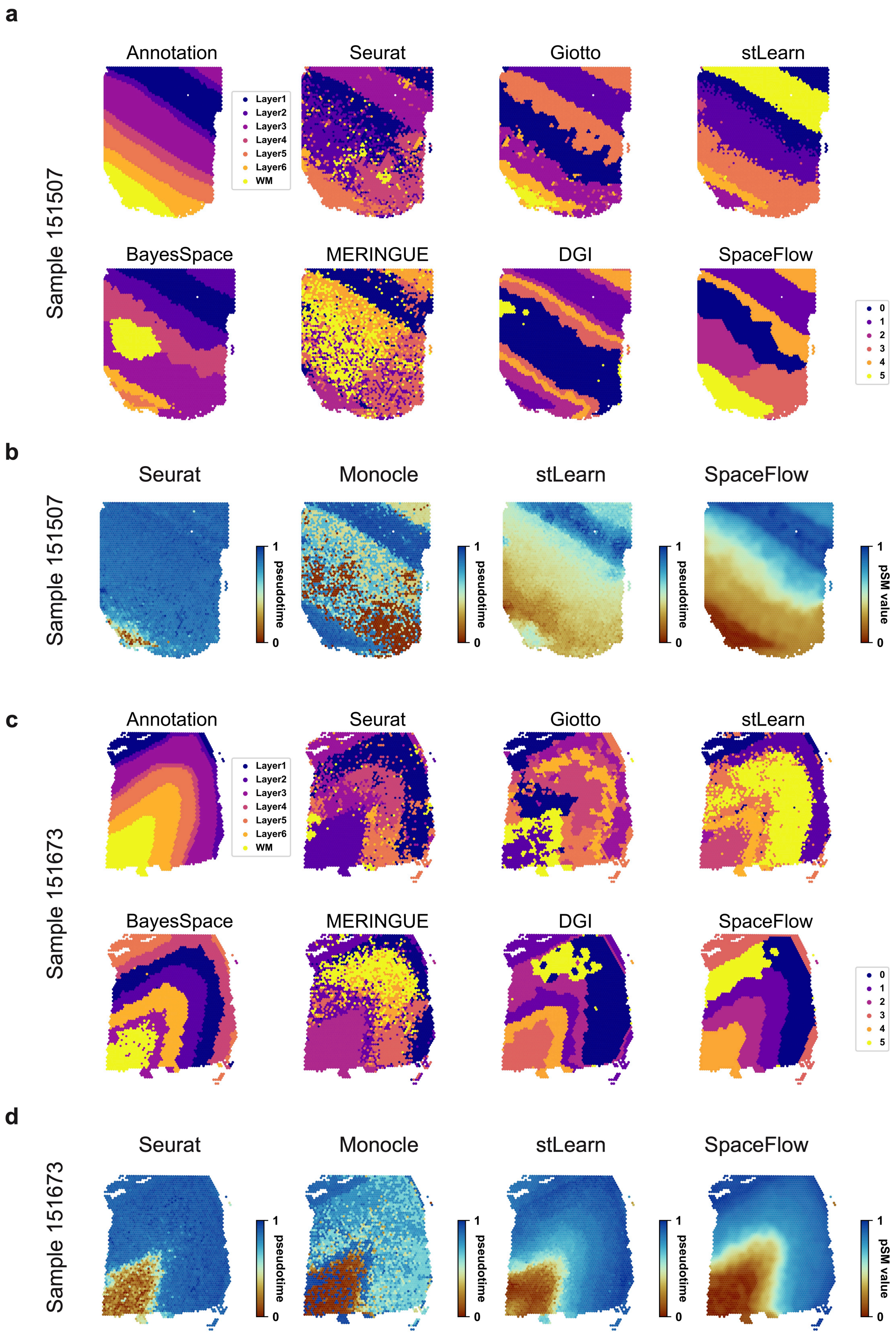

Supplementary Figure 1. Benchmarking on sections 151507 and 151673 using LIBD human dorsolateral prefrontal cortex (DLPFC) ST data [1]. a. Domain annotation (top left panel) and segmentations given by different methods (other panels) using section 151507 of DLPFC data. **b**. The spatial visualization of pseudotimes calculated by Seurat, Monocle, stLearn, and the pseudo-Spatiotemporal Map (pSM) generated by SpaceFlow on section 151507 of DLPFC data. **c.** Domain annotation (top left panel) and segmentations given by different methods (other panels) using section 151673 of DLPFC data. **d.** The spatial visualization of pseudotimes calculated by Seurat, Monocle, stLearn, and the pSM generated by SpaceFlow on section 151673 of DLPFC data.

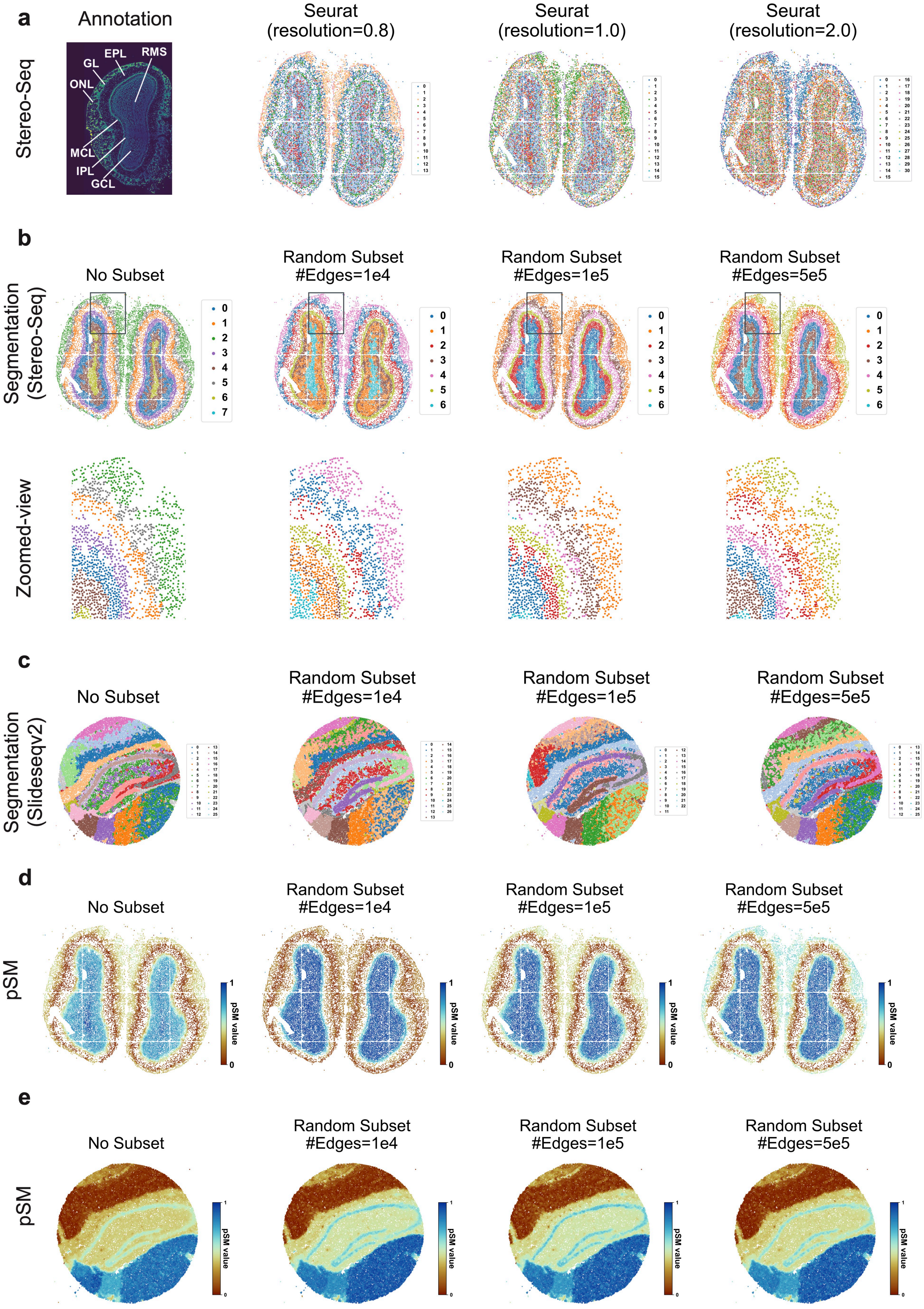
a
Annotation
Stereo-Seq
GL
EPL
RMS
ONL
MCL
IPL
GCL
Seurat (resolution=0.8)
Seurat (resolution=1.0)
Seurat (resolution=2.0)
b
No Subset
Random Subset #Edges=1e4
Random Subset #Edges=1e5
Random Subset #Edges=5e5
Segmentation (Stereo-Seq)
Zoomed-view
c
Segmentation (Slideseqv2)
No Subset
Random Subset #Edges=1e4
Random Subset #Edges=1e5
Random Subset #Edges=5e5
d
pSM
No Subset
Random Subset #Edges=1e4
Random Subset #Edges=1e5
Random Subset #Edges=5e5
pSM value
e
pSM
No Subset
Random Subset #Edges=1e4
Random Subset #Edges=1e5
Random Subset #Edges=5e5
pSM value

Supplementary Figure 2. Domain segmentation and pSM on Stereo-seq data and Slide-seqv2 data. a. Domain annotation (left panel) and segmentations given by Seurat with different resolution settings (right three panels) on Stereo-seq data. **b-c**. Domain segmentations of (**b**) Stereo-seq data and (**c**) Slide-seqv2 data given by SpaceFlow computing regularization over different subsets of cells, showing lack of meaningful variation even when regularization is computed over only a subset. **d-e.** The pseudo-Spatiotemporal Map (pSM) of (**d**) Stereo-seq data and (**e**) Slide-seqv2 data given by SpaceFlow computing regularization over different subsets of cells, again showing qualitatively matching results in each case.

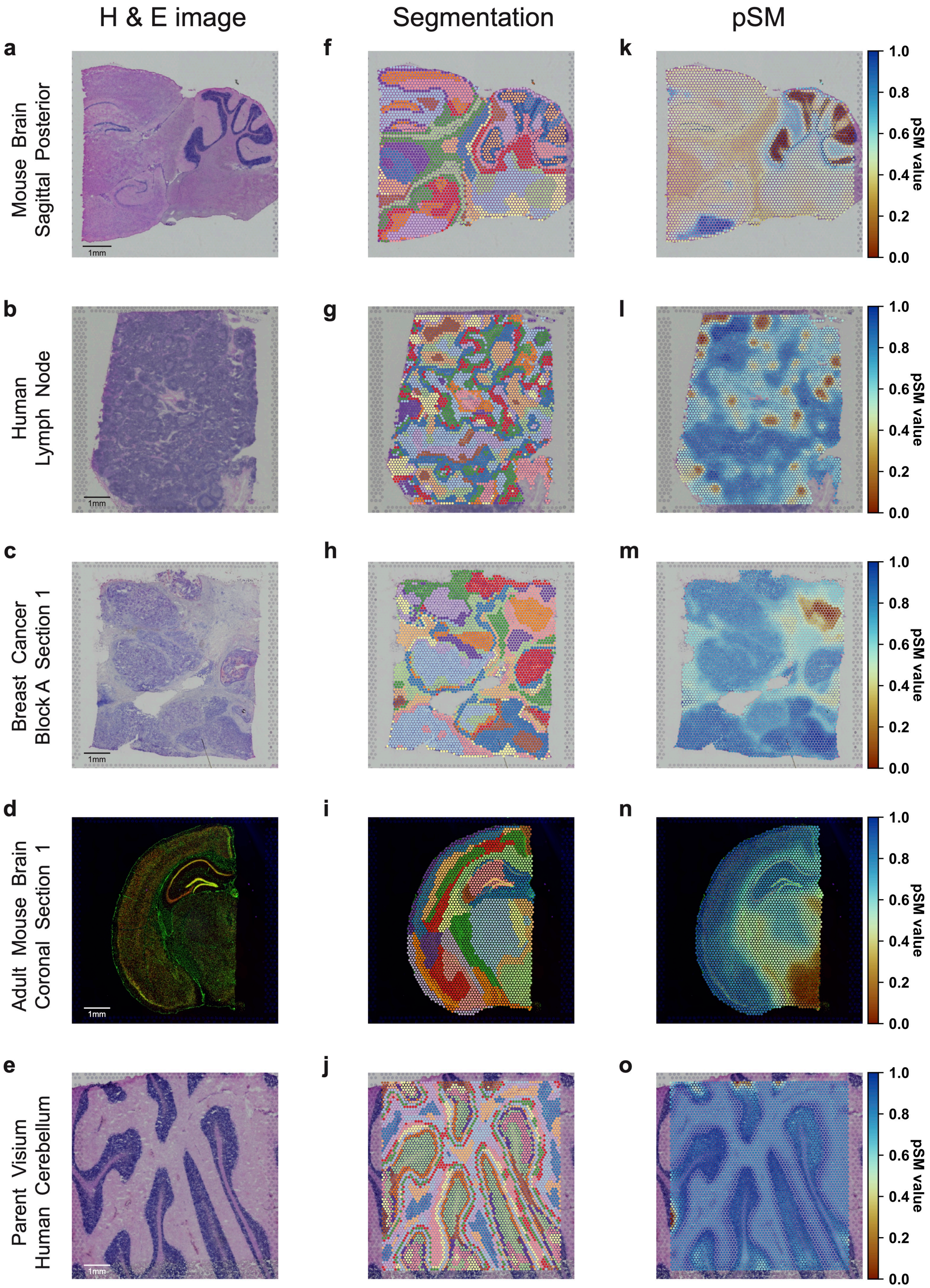

H & E image
Segmentation
pSM
a
b
c
d
e
f
g
h
i
j
k
l
m
n
o
Mouse Brain
Sagittal Posterior
Human
Lymph Node
Breast Cancer
Block A Section 1
Adult Mouse Brain
Coronal Section 1
Parent Visium
Human Cerebellum
1mm
pSM value
1.0
0.8
0.6
0.4
0.2
0.0

Supplementary Figure 3. Domain segmentation and pSM on five 10x Genomics official Visium datasets. a-e. H & E images of five Visium datasets (**a**. V1 Mouse Brain Sagittal Posterior, **b**. V1 Human Lymph Node, **c**. V1 Breast Cancer Block A Section 1, **d**. V1 Adult Mouse Brain Coronal Section 1, **e**. Parent Visium Human Cerebellum). **f-j.** Domain segmentations of the corresponding datasets given by SpaceFlow. **k-o**. pSM of the five datasets given by SpaceFlow.

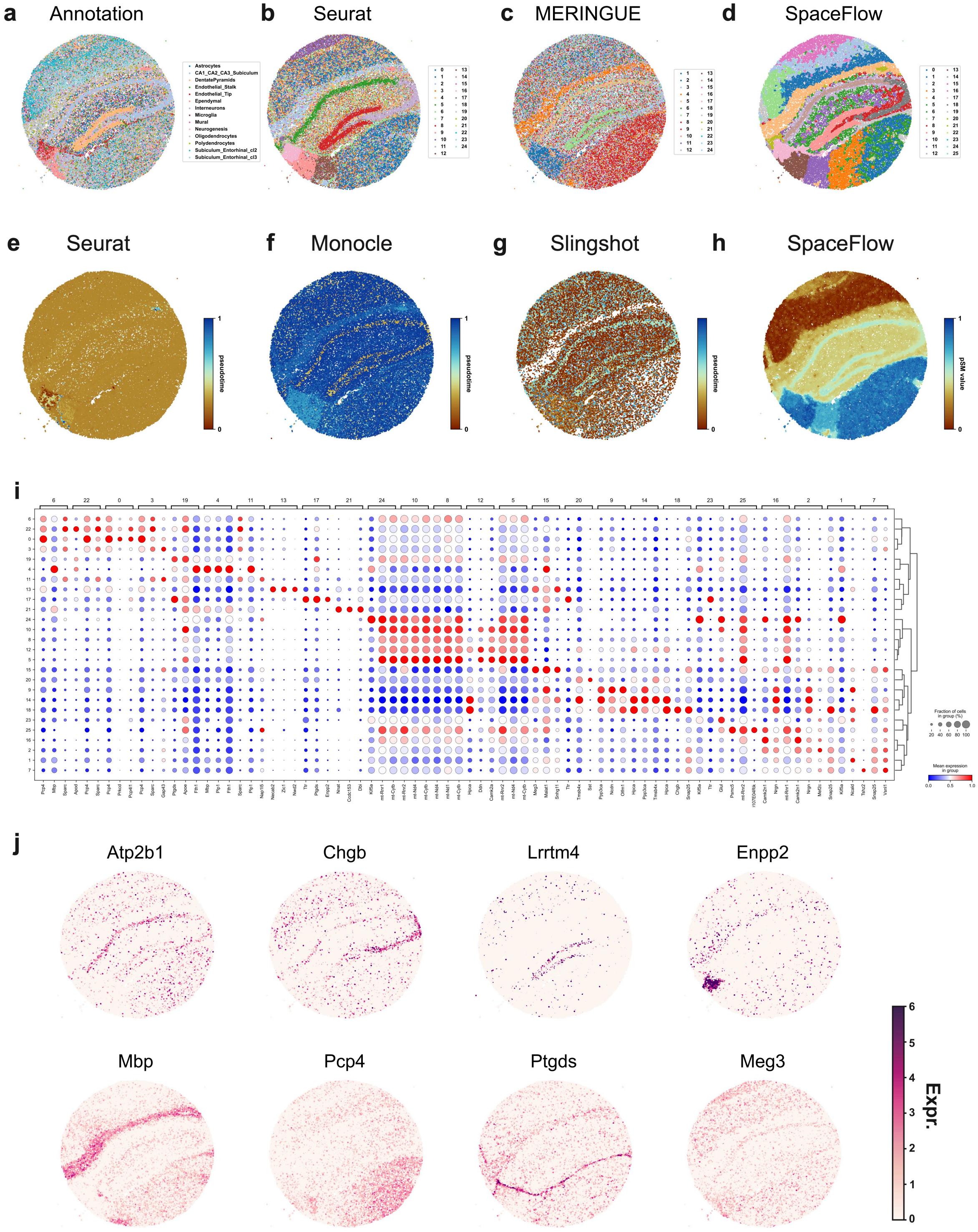
a
Annotation
Astrocytes
CA1_CA2_CA3_Subiculum
DentatePyramids
Endothelial_Stalk
Endothelial_Tip
Ependymal
Interneurons
Microglia
Mural
Neurogenesis
Oligodendrocytes
Polydendrocytes
Subiculum_Entorhinal_cl2
Subiculum_Entorhinal_cl3
b
Seurat
c
MERINGUE
d
SpaceFlow
e
Seurat
pseudotime
f
Monocle
pseudotime
g
Slingshot
pseudotime
h
SpaceFlow
pSM value
i
Fraction of cells in group (%)
Mean expression in group
j
Atp2b1
Chgb
Lrrtm4
Enpp2
Mbp
Pcp4
Ptgds
Meg3
Expr.

Supplementary Figure 4. SpaceFlow analysis on slideseq v2 dataset [2]**. a-** Annotation **b-d**. Domain segmentations produced by Seurat (**b**), MERINGUE (**c**) and SpaceFlow (**d**). **e-h**. The spatial visualization of pseudotimes calculated by Seurat (**e**), Monocle (**f**), Slingshot (**g**), and the pSM generated by SpaceFlow (**h**). **i**. Dot plot of the gene expression of domain-specific markers. The dot size represents the fraction of cells in a domain expressing the marker and the color intensity represents the average expression of the marker in that domain. **j.** Spatial expression for representative markers of the identified domains.

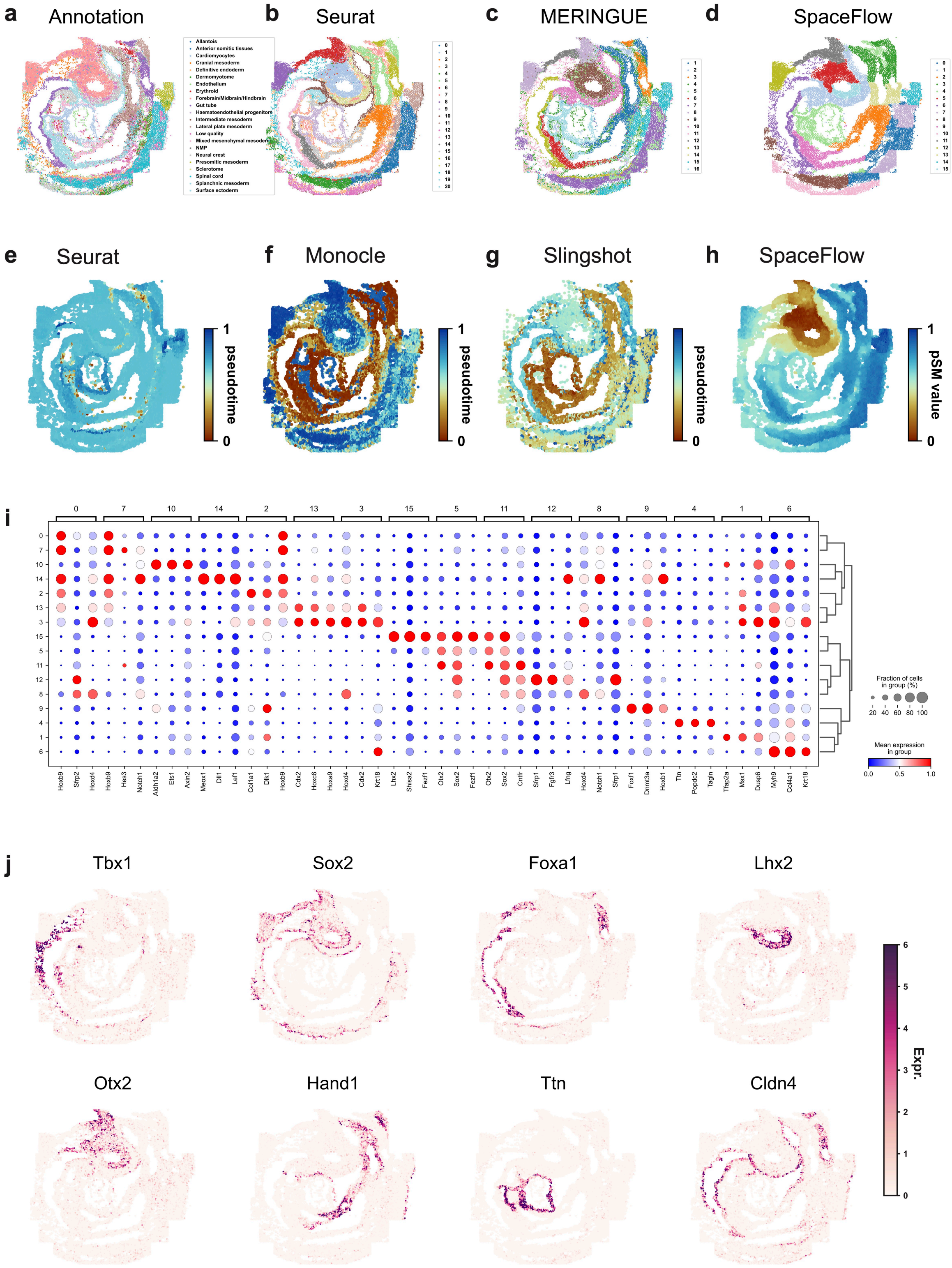
a
Annotation
Allantois
Anterior somitic tissues
Cardiomyocytes
Cranial mesoderm
Definitive endoderm
Dermomyotome
Endothelium
Erythroid
Forebrain/Midbrain/Hindbrain
Gut tube
Haematoendothelial progenitors
Intermediate mesoderm
Lateral plate mesoderm
Low quality
Mixed mesenchymal mesoderm
NMP
Neural crest
Presomitic mesoderm
Sclerotome
Spinal cord
Splanchnic mesoderm
Surface ectoderm
b
Seurat
c
MERINGUE
d
SpaceFlow
e
Seurat
f
Monocle
g
Slingshot
h
SpaceFlow
pseudotime
pSM value
i
Fraction of cells in group (%)
20 40 60 80 100
Mean expression in group
0.0 0.5 1.0
Hoxb9 Sfrp2 Hoxd4 Hoxb9 Hes3 Notch1 Aldh1a2 Ets1 Axin2 Meox1 Dll1 Lef1 Col1a1 Dlk1 Hoxb9 Cdx2 Hoxc6 Hoxa9 Hoxd4 Cdx2 Krt18 Lhx2 Shisa2 Fezf1 Otx2 Sox2 Fezf1 Otx2 Sox2 Cntfr Sfrp1 Fgf3 Lfng Hoxd4 Notch1 Sfrp1 Foxf1 Dnmt3a Hoxb1 Ttn Popdc2 Tagln Tfap2a Msx1 Dusp6 Myh9 Col4a1 Krt18
j
Tbx1
Sox2
Foxa1
Lhx2
Otx2
Hand1
Ttn
Cldn4
Expr.

Supplementary Figure 5. SpaceFlow analysis on seqFISH mouse embryogenesis dataset [3]**. a-** Annotation **b-d**. Domain segmentations produced by Seurat (**b**), MERINGUE (**c**) and SpaceFlow (**d**). **e-h**. The spatial visualization of pseudotimes calculated by Seurat (**e**), Monocle (**f**), Slingshot (**g**), and the pSM generated by SpaceFlow (**h**). **i**. Dot plot of the gene expression of domain-specific markers. The dot size represents the fraction of cells in a domain expressing the marker and the color intensity represents the average expression of the marker in that domain. **j.** Spatial expression for representative markers of the identified domains.

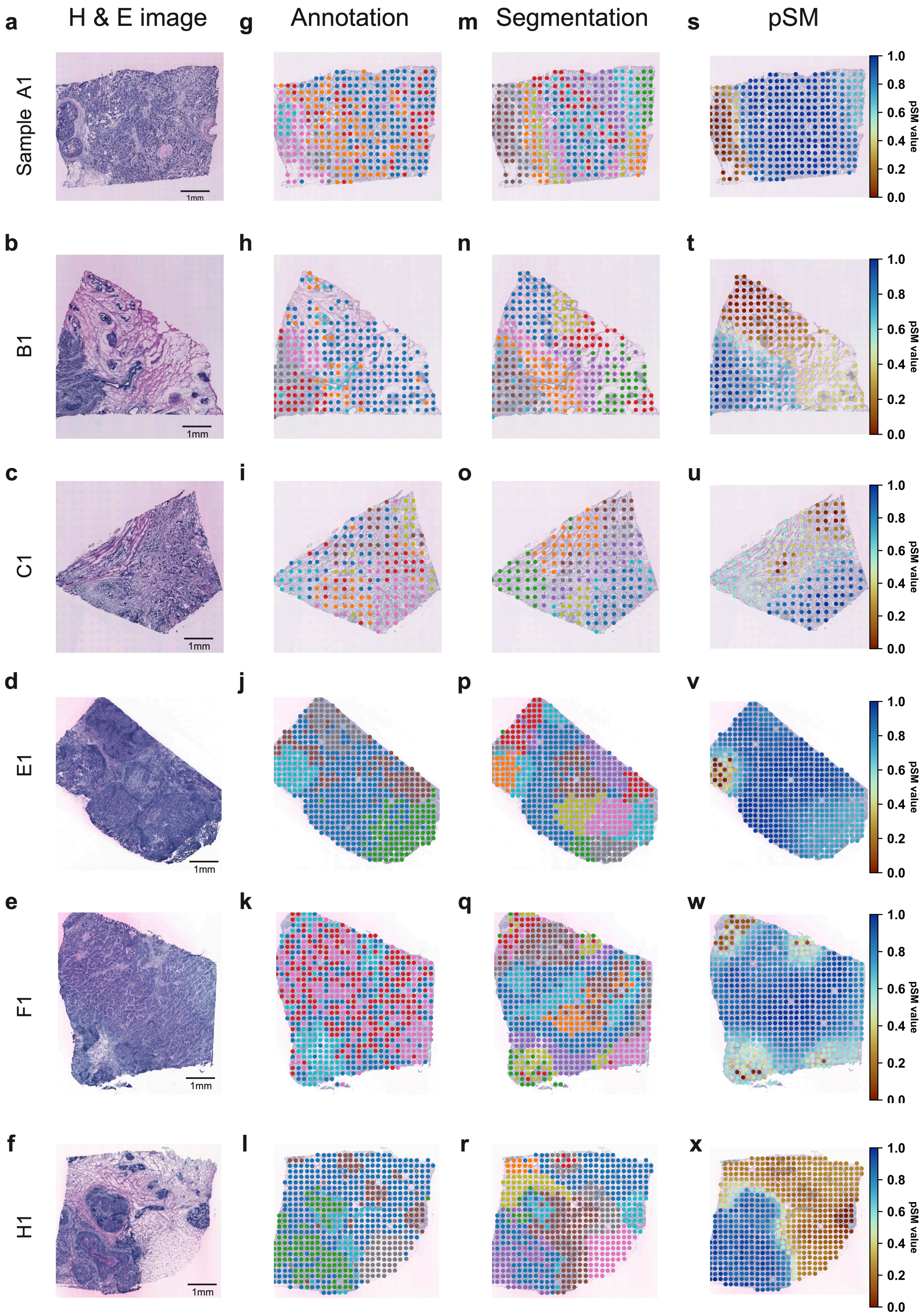
a
H & E image
g
Annotation
m
Segmentation
s
pSM
Sample A1
B1
C1
E1
F1
H1
1mm
pSM value
1.0
0.8
0.6
0.4
0.2
0.0
b
c
d
e
f
h
i
j
k
l
n
o
p
q
r
t
u
v
w
x

Supplementary Figure 6. Domain segmentation and pSM on six samples of Human Breast Cancer datasets [4]. a-f. H & E images of the six samples (Sample A1, B1, C1, E1, F1, H1, respectively). **g-l.** Annotations of the datasets given by the original paper. **m-r.** Domain segmentations of the datasets given by SpaceFlow. **s-x**. pSM of the datasets given by SpaceFlow.

Supplementary Tables

Supplementary Table 1. Functional Roles of Ligand Receptors showed cell-cell communications in human breast cancer progression

Ligand/Receptors	Function in Tumor Progression
COL1A1, COL1A2, COL6A1, COL6A2, COL6A3, COL4A2	As components of the extracellular matrix (ECM), and has been shown to promote invasion, metastasis, proliferation, tumorigenesis, death resistance of cancer cells, and regulate anti-tumor immunity, hypoxic condition. Cancer cells also can reversely reshape collagen to construct a positive feedback loop, which gradually encourages cancer progression.[5]
SDC1	Facilitate breast cancer metastasis.[6,7]
SDC4	The upregulation contributes to epithelial to mesenchymal transition (EMT) of cancer cells.[8]
CD44	The activation result in the activation of signaling pathways that induce cell proliferation and survival, regulate cytoskeletal changes, and encourage cellular motility. CD44 may also play roles in EMT and the adaptive plasticity of cancer cells.[9]
APP	Promote the migration and invasion of breast cancer cells by regulating the MAPK signaling pathway.[10]
CD74	Interact with CD44 and promote tumorigenesis and metastasis via RHOA-mediated cofilin phosphorylation in human breast cancer cells.[11]
MDK	A key player in cancer progression.[12]

Reference

1. Maynard, K. R. *et al.* Transcriptome-scale spatial gene expression in the human dorsolateral prefrontal cortex. *Nat. Neurosci.* **24**, 425–436 (2021).
2. Stickels, R. R. *et al.* Highly sensitive spatial transcriptomics at near-cellular resolution with Slide-seqV2. *Nat. Biotechnol.* **39**, 313–319 (2021).
3. Lohoff, T. *et al.* Integration of spatial and single-cell transcriptomic data elucidates mouse organogenesis. *Nat. Biotechnol.* **40**, 74–85 (2022).
4. Andersson, A. *et al.* Spatial deconvolution of HER2-positive breast cancer delineates tumor-associated cell type interactions. *Nat. Commun.* **12**, 6012 (2021).
5. Xu, S. *et al.* The role of collagen in cancer: from bench to bedside. *J. Transl. Med.* **17**, 309 (2019).
6. Chute, C. *et al.* Syndecan-1 induction in lung microenvironment supports the establishment of breast tumor metastases. *Breast Cancer Res.* **20**, (2018).
7. Sayyad, M. R. *et al.* Syndecan-1 facilitates breast cancer metastasis to the brain. *bioRxiv* (2019) doi:10.1101/565648.
8. Toba-Ichihashi, Y., Yamaoka, T., Ohmori, T. & Ohba, M. Up-regulation of Syndecan-4 contributes to TGF-β1-induced epithelial to mesenchymal transition in lung adenocarcinoma A549 cells. *Biochem. Biophys. Rep.* **5**, 1–7 (2016).
9. Chen, C., Zhao, S., Karnad, A. & Freeman, J. W. The biology and role of CD44 in cancer progression: therapeutic implications. *J. Hematol. Oncol.* **11**, (2018).
10. Wu, X., Chen, S. & Lu, C. Amyloid precursor protein promotes the migration and invasion of breast cancer cells by regulating the MAPK signaling pathway. *Int. J. Mol. Med.* **45**, 162–174 (2020).
11. Liu, Z. *et al.* CD74 interacts with CD44 and enhances tumorigenesis and metastasis via RHOA-mediated cofilin phosphorylation in human breast cancer cells. *Oncotarget* **7**, 68303–68313 (2016).
12. Filippou, P. S., Karagiannis, G. S. & Constantinidou, A. Midkine (MDK) growth factor: a key player in cancer progression and a promising therapeutic target. *Oncogene* **39**, 2040–2054 (2020).
