# Supplementary material for: Identifying multicellular spatiotemporal organization of cells with SpaceFlow
Source: Nat Commun. 2022 Jul 14;13:4076. doi: 10.1038/s41467-022-31739-w (PMC9283532; doi:10.1038/s41467-022-31739-w)
Supplement: Supplementary file 3 — Description of additional Supplementary File [file 41467_2022_31739_MOESM3_ESM.pdf]

Descriptions of Additional Supplementary Data Files

Supplementary Dataset 1 : Supplementary excel file lists marker genes for domains identified by SpaceFlow using chicken development spatial transcriptome data. Related to Figure 4.

Supplementary Dataset 2: Supplementary excel file lists marker genes for domains identified by SpaceFlow using human breast cancer spatial transcriptome data in Figure 5 (sample G1).
