# Supplementary material for: Identifying multicellular spatiotemporal organization of cells with SpaceFlow
Source: Nat Commun. 2022 Jul 14;13:4076. doi: 10.1038/s41467-022-31739-w (PMC9283532; doi:10.1038/s41467-022-31739-w)
Supplement: Supplementary file 6 — Reporting Summary [file 41467_2022_31739_MOESM6_ESM.pdf]

nature portfolio

Corresponding author(s): Qing Nie
Last updated by author(s): Jun 20, 2022

Reporting Summary

Nature Portfolio wishes to improve the reproducibility of the work that we publish. This form provides structure for consistency and transparency in reporting. For further information on Nature Portfolio policies, see our Editorial Policies and the Editorial Policy Checklist.

Statistics

For all statistical analyses, confirm that the following items are present in the figure legend, table legend, main text, or Methods section.

n/a	Confirmed	
☐	☒	The exact sample size (*n*) for each experimental group/condition, given as a discrete number and unit of measurement
☒	☐	A statement on whether measurements were taken from distinct samples or whether the same sample was measured repeatedly
☒	☐	The statistical test(s) used AND whether they are one- or two-sided<br>*Only common tests should be described solely by name; describe more complex techniques in the Methods section.*
☒	☐	A description of all covariates tested
☒	☐	A description of any assumptions or corrections, such as tests of normality and adjustment for multiple comparisons
☒	☐	A full description of the statistical parameters including central tendency (e.g. means) or other basic estimates (e.g. regression coefficient) AND variation (e.g. standard deviation) or associated estimates of uncertainty (e.g. confidence intervals)
☒	☐	For null hypothesis testing, the test statistic (e.g. *F*, *t*, *r*) with confidence intervals, effect sizes, degrees of freedom and *P* value noted<br>*Give P values as exact values whenever suitable.*
☒	☐	For Bayesian analysis, information on the choice of priors and Markov chain Monte Carlo settings
☒	☐	For hierarchical and complex designs, identification of the appropriate level for tests and full reporting of outcomes
☒	☐	Estimates of effect sizes (e.g. Cohen's *d*, Pearson's *r*), indicating how they were calculated

Our web collection on statistics for biologists contains articles on many of the points above.

Software and code

Policy information about availability of computer code

Data collection	No software was used for data collection.
Data analysis	We performed the data analysis with newly developed package SpaceFlow in this manuscript, which is available at https://github.com/hongleir/SpaceFlow. SpaceFlow was developed in Python 3.7 with a package dependency on Pytorch v1.10.0. The marker gene identification analysis is performed using Scanpy v1.8.2 package. Gene Ontology (GO) Enrichment Analysis is carried out in the GO Consortium website (http://geneontology.org/). The cell-cell communication inference is performed through CellChat v1.1.3 in a R v4.1.2 environment. Seurat v4, Giotto, stLearn, MERINGUE, BayesSpace are used in benchmarking analysis.

For manuscripts utilizing custom algorithms or software that are central to the research but not yet described in published literature, software must be made available to editors and reviewers. We strongly encourage code deposition in a community repository (e.g. GitHub). See the Nature Portfolio guidelines for submitting code & software for further information.

Data

Policy information about availability of data

All manuscripts must include a data availability statement. This statement should provide the following information, where applicable:

- Accession codes, unique identifiers, or web links for publicly available datasets
- A description of any restrictions on data availability
- For clinical datasets or third party data, please ensure that the statement adheres to our policy

All data analyzed in this paper can be downloaded in raw form from the original publication. Specifically, the DLPFC data is available in the spatialLIBD package (http://spatial.libd.org/spatialLIBD). The processed Stereo-seq data from mouse olfactory bulb tissue is accessible on https://github.com/JinmiaoChenLab/SEDR_analyses. The chicken heart ST data is retrieved from GEO database (GSE149457[https://www.ncbi.nlm.nih.gov/geo/query/acc.cgi?acc=GSE149457]). The

human breast cancer ST data can be obtained from https://zenodo.org/record/4751624.

The Slide-seq V2 can be accessed in Squidpy package or downloaded from https://singlecell.broadinstitute.org/single_cell/study/SCP815/highly-sensitive-spatial-transcriptomics-at-near-cellular-resolution-with-slide-seqv2. The seqFISH data can be accessed at https://marionilab.cruk.cam.ac.uk/SpatialMouseAtlas/. The Gene Ontology Consortium databased can be accessed via http://geneontology.org/.

Field-specific reporting

Please select the one below that is the best fit for your research. If you are not sure, read the appropriate sections before making your selection.

- [x] Life sciences
- [ ] Behavioural & social sciences
- [ ] Ecological, evolutionary & environmental sciences

For a reference copy of the document with all sections, see nature.com/documents/nr-reporting-summary-flat.pdf

Life sciences study design

All studies must disclose on these points even when the disclosure is negative.

Sample size	No biological experiment was conducted in this study. The public LIBD human dorsolateral prefrontal cortex (DLPFC) ST dataset we used has sample sizes are greater than previous publications with the earlier Spatial Transcriptomics technology that preceded Visium (DOIs: 10.1126/science.aaf2403 and 10.1126/science.aav9776). Therefore, the sample size is sufficient.
Data exclusions	Spatial Transcriptome datasets with smaller size are more sensitive to the noise in measurements and it is common to remove low-quality cells. In this study, cells with expression of fewer than 100 genes are removed for more robust analysis. These details have been described in Methods, and we provided the scripts to reproduce the data exclusions process.
Replication	No biological experiment was conducted in this study. For computational tasks, we run the program on spatially-adjacent replicates from public LIBD human dorsolateral prefrontal cortex (DLPFC) ST data, and the results were reproducible on different replicates. We uploaded the scripts to reproduce the results in this study to https://github.com/hongleir/SpaceFlow.
Randomization	The allocation was random.
Blinding	All results are based on published data which have been studied in their original publications. Therefore, blinding from investigators is not possible when we reanalyzed the data. Group allocation information was never provided to the computational algorithms.

Reporting for specific materials, systems and methods

We require information from authors about some types of materials, experimental systems and methods used in many studies. Here, indicate whether each material, system or method listed is relevant to your study. If you are not sure if a list item applies to your research, read the appropriate section before selecting a response.

Materials & experimental systems

n/a	Involved in the study	
☒	☐	Antibodies
☒	☐	Eukaryotic cell lines
☒	☐	Palaeontology and archaeology
☒	☐	Animals and other organisms
☒	☐	Human research participants
☒	☐	Clinical data
☒	☐	Dual use research of concern

Methods

n/a	Involved in the study	
☒	☐	ChIP-seq
☒	☐	Flow cytometry
☒	☐	MRI-based neuroimaging
